# Role of Type I Interferon Signaling and Microglia in the Abnormal Long-term Potentiation and Object Place Recognition Deficits of Male Mice With a Mutation of the Tuberous Sclerosis 2 Gene

**DOI:** 10.1016/j.bpsgos.2022.03.015

**Published:** 2022-04-14

**Authors:** Manuel F. López-Aranda, Gayle M. Boxx, Miranda Phan, Karen Bach, Rochelle Mandanas, Isaiah Herrera, Sunrae Taloma, Chirag Thadani, Odilia Lu, Raymond Bui, Shuhan Liu, Nan Li, Yu Zhou, Genhong Cheng, Alcino J. Silva

**Affiliations:** a Departments of Neurobiology, Psychology, and Psychiatry, University of California, Los Angeles, Los Angeles, California; b Integrative Center for Learning and Memory, University of California, Los Angeles, Los Angeles, California; c Brain Research Institute, University of California, Los Angeles, Los Angeles, California; d Department of Microbiology, Immunology and Molecular Genetics, University of California, Los Angeles, Los Angeles, California; e Department of Physiology and Pathophysiology, School of Basic Medical Sciences, Qingdao University, Qingdao, China; f Institute of Brain Function and Diseases, Qingdao University, Qingdao, China

**Keywords:** Hippocampal memory, LTP, Microglia, OPR, Tsc2, Type I IFN

## Abstract

**Background:**

Tuberous sclerosis complex is a genetic disorder associated with high rates of intellectual disability and autism. Mice with a heterozygous null mutation of the *Tsc2* gene (*Tsc2*^+/−^) show deficits in hippocampal-dependent tasks and abnormal long-term potentiation (LTP) in the hippocampal CA1 region. Although previous studies focused on the role of neuronal deficits in the memory phenotypes of rodent models of tuberous sclerosis complex, the results presented here demonstrate a role for microglia in these deficits.

**Methods:**

To test the possible role of microglia and type I interferon in abnormal hippocampal-dependent memory and LTP of *Tsc2*^+/−^ mice, we used field recordings in CA1 and the object place recognition (OPR) task. We used the colony stimulating factor 1 receptor inhibitor PLX5622 to deplete microglia in *Tsc2*^+/−^ mice and interferon alpha/beta receptor alpha chain null mutation (*Ifnar1*^−/−^) to manipulate a signaling pathway known to modulate microglia function.

**Results:**

Unexpectedly, we demonstrate that male, but not female, *Tsc2*^+/−^ mice show OPR deficits. These deficits can be rescued by depletion of microglia and by the *Ifnar1*^−/−^ mutation. In addition to rescuing OPR deficits, depletion of microglia also reversed abnormal LTP of the *Tsc2*^+/−^ mice. Altogether, our results suggest that altered IFNAR1 signaling in microglia causes the abnormal LTP and OPR deficits of male *Tsc2*^+/−^ mice.

**Conclusions:**

Microglia and IFNAR1 signaling have a key role in the hippocampal-dependent memory deficits and abnormal hippocampal LTP of *Tsc2*^+/−^ male mice.

Tuberous sclerosis complex (TSC) is a genetic condition caused by mutations in either the *TSC1* (1) or *TSC2* (2) gene. Autism spectrum disorder (3, 4, 5, 6) and cognitive deficits (7, 8, 9) are common in TSC. Prior studies have suggested that humans (8,10), as well as *Tsc1* (7,11) and *Tsc2* (9,12) rodent models of TSC, show hippocampal-dependent spatial memory deficits, as assessed in the Morris water maze (7,9,12) and radial arm maze (9). In addition, other forms of hippocampal-dependent memory, including contextual fear memory (7,9,11,12), are impaired in TSC mouse models. Previous studies suggested that abnormal hippocampal long-term potentiation (LTP) may underlie these memory deficits (9,13), thus suggesting that neuronal deficits have a principal role in the memory phenotypes of rodent models of TSC (14,15).

The object place recognition (OPR) task is designed to assess spatial memory and discrimination. This test is based on a rodent’s tendency to spend more time investigating a familiar object that has been moved to a new location (16,17). Previous studies showed that lesions (16,18), pharmacological antagonists (19), and genetic manipulations (20) directed at the hippocampus resulted in impairments in OPR, demonstrating a role for the hippocampus in this task (16,18, 19, 20, 21). Recently, we demonstrated that microglia and type I interferon (IFN) play a critical role in the social memory deficits of *Tsc2*^+/−^ mice triggered by early postnatal immune activation (22). Here, we used the OPR task to test the hypothesis that microglia and type I IFN signaling play a role in hippocampal-dependent memory deficits and abnormal hippocampal LTP of *Tsc2*^+/−^ mice.

## Methods and Materials

The Chancellor’s Animal Research Committee at the University of California Los Angeles approved the research protocols used here.

### Experimental Design and Subject Details

#### *Tsc2*^+/−^ Mice

We first crossed male *Tsc2*^+/−^ mice (23) with female wild-type (WT) mice. *Tsc2*^+/−^ male breeders were on a C57BL/6Ncrl genetic background (Charles River Laboratories, Cat. # 027). We used C57BL/6J females (JAX, Cat. # 000664) to generate experimental mice. Pregnant females were single housed and left undisturbed except for weekly cage changes. Pregnancy was determined by checking for abdominal distension. For all of the mice studied here, pregnant females were checked every day to determine the exact day when the pups were born (designated postnatal day [P] 0). Tail biopsies for genotyping were taken around P40.

#### *Ifnar1*^−/−^// *Tsc2*^+/−^ Mice

To test the role of type I IFN alpha and beta in the phenotype of male *Tsc2*^+/−^ mice, we generated mice that included both *Tsc2*^+/−^ and *Ifnar1*^−/−^ null mutations by crossing *Tsc2*^+/−^ male breeders with *Ifnar1*^−/−^ females (bred on a C57BL/6J background) followed by intercrosses. Tail biopsies for genotyping were taken around P40.

#### *Cx3cr1*^Cre^–*Tsc2*^Flox^ Mice

To confirm whether microglia play a critical role in the hippocampal-dependent memory deficits that male *Tsc2*^+/−^ mice show, we crossed *Cx3cr1*^**Cre**^ mice (JAX stock #025524) with *Tsc2*^Flox^ mice (JAX stock #027458) to generate mice that carry the *Tsc2* mutation only in microglia. Tail biopsies for genotyping were taken around P40.

### Method Details

#### OPR Test

Before the test, mice were handled for 8 minutes daily for 6 consecutive days. Then, in the next 2 days, they were habituated in the OPR open field (41.5 × 41.5 × 40.5 cm) for 12 minutes each day. During the training session, mice were placed back in the OPR open field, this time with 2 identical objects, and they were allowed to explore freely for 7 minutes. After either a 1-hour or 24-hour interval, mice were tested for OPR with the previously presented objects, one of them in a new location. The object in the new location during the test was counterbalanced between trials. The open field was cleaned after each session using 70% ethanol. Mice were trained or tested once per day. Sessions were video recorded and scored offline by either 1 or 2 observers, as described above. The variation between observers was normally <2 seconds in the 7-minute exploration sessions. Exploration was counted only when the test mouse touched one of the objects with its nose. All experiments and scoring were carried out blinded to genotype and treatment condition. The objects included in this study were small bottles or containers of different shapes.

#### Novel Object Recognition Test

The novel object recognition test was carried out as described previously (24), 24 hours before or after OPR (to counterbalance possible order effects). During the training session, mice were placed in the novel object recognition open field (same as the OPR open field) with 2 identical objects, and they were allowed to explore freely for 7 minutes. After a 24-hour interval, mice were tested for object memory with the previously presented object and a new object. Location of the new object during the test was counterbalanced between trials. Mice were trained or tested once per day. The open field was cleaned after each session using 70% ethanol. Sessions were video recorded and scored offline as described above. The variation between observers was normally <2 seconds in the 7-minute exploration sessions. Exploration was counted only when the test mouse touched the objects with its nose. All experiments and scoring were carried out blinded to genotype and treatment condition. The objects included in this study were small bottles or containers of different shapes.

#### Rapamycin Treatment

Rapamycin (5 mg/kg; LC Laboratories, Cat. # R-5000) was freshly dissolved in vehicle solution (100% DMSO; Sigma-Aldrich, Cat. # D5879-500ML) before use. Adult (4–6 months old) *Tsc2*^+/−^ mice were treated with a single intraperitoneal injection of rapamycin (5 mg/kg) or vehicle (DMSO) daily for 5 days prior to the OPR test. Mice were tested for OPR 18 hours after the last injection of rapamycin or DMSO as well as 2 months later. Because 100% DMSO could have an impact, we monitored that possibility in our studies. We observed that the administration of 100% DMSO did not lead to deficits in social interaction in the *Tsc2*^+/−^ mice. In addition, we did not observe any deficits in locomotion or exploration in the *Tsc2* mice injected with DMSO compared with mice not injected.

#### PLX5622 Treatment

PLX5622 (PLX) and control rodent diet was provided by Plexxikon Inc. and formulated in AIN-76A standard chow by Research Diets. Adult (4–6 months old) *Tsc2*^+/−^ mice were fed with PLX (1200 mg/kg; Cat. # D11100404i) or control chow (Cat. # D10001i) for 21 days (25). At day 21 of PLX treatment, mice were tested for OPR. Mice were tested again in the OPR test 2 months after the last day of PLX treatment.

#### Polyinosinic:Polycytidylic Acid Administration

Poly(I:C) (polyinosinic:polycytidylic acid) potassium salt [Sigma, Cat. # P9582-50MG; poly(I:C) is supplied at 10% of the total weight of the salt; dosage was based on the weight of poly(I:C) itself] was dissolved in vehicle solution (0.9% sterile saline) before use. *Tsc2*^+/−^ mice were injected intraperitoneally with poly(I:C) (20 mg/kg) or vehicle at P3, P7, and P14.

#### Immunohistochemistry

Immunohistochemistry was performed as described earlier (26). Briefly, mice were perfused transcardially with a fixative containing 4% paraformaldehyde and cryoprotected with 30% sucrose. Coronal and sagittal brain sections 60 μm thick were incubated overnight at 4 °C with monoclonal rabbit anti-Iba1 (1:1000 dilution; Wako Chemicals, Cat. # 019-19741). The sections were then incubated for 90 minutes with Alexa Fluor 488 goat anti-rabbit IgG (immunoglobulin G) (1:500 dilution; Invitrogen, Cat. # A11011). After that, the sections were incubated in DAPI (1:1000 dilution) for 15 minutes and then in phosphate-buffered saline for 14 minutes. Immunofluorescence labeling was detected with a confocal microscope.

#### Hippocampal Slice Physiology

Coronal hippocampal slices (350 μm in thickness) were freshly made with a Leica VT-1000 vibratome in ice-cold cutting solution containing 7 mM MgSO_4_, 1 mM CaCl_2_, 2.5 mM KCl, 26 mM NaHCO_3_, 1 mM NaH_2_PO_4_, 30-mM glucose, 1.3 mM sodium L-ascorbate, 1 mM kynurenic acid, 3 mM sodium pyruvate, and 119 mM choline chloride. Slices were recovered in a solution consisting of 85 mM NaCl, 2.5 mM KCl, 4 mM MgCl_2_, 0.5 mM CaCl_2_, 1.25 mM NaH_2_PO_4_, 24 mM NaHCO_3_, 25-mM glucose, and 50 mM sucrose for at least 1 hour at room temperature. Slices were then transferred to the submerged recording chamber and continuously perfused with artificial cerebrospinal fluid (30 °C) at a rate of approximately 3 mL/min. Artificial cerebrospinal fluid contains 120 mM NaCl, 3.5 mM KCl, 1.3 mM MgCl_2_, 2.5 mM CaCl_2_, 1.25 mM NaH_2_PO_4_, 26 mM NaHCO_3_, and 10 mM D-glucose. All solutions (pH 7.2∼7.4, Osmo 290∼310) were oxygenated with 95% O_2_/5% CO_2_ mixture gas.

Field excitatory postsynaptic potentials (fEPSPs) in the Schaffer collateral-CA1 in the dorsal hippocampus were evoked with an FHC bipolar platinum microelectrode (9). The input-output curve was constructed by varying stimuli intensity (∼ 10-100 μA) while measuring the corresponding presynaptic volley and the initial fEPSP slope. LTP of Schaffer collateral-CA1 synapses was induced by a 100-Hz (1 second) tetanus. All stimulation pulses were 100 μs in duration and at approximately one third to one half of the intensity that induces a maximal fEPSP response. Data were obtained using MultiClamp 700B amplifier equipped with a Digidata 1440A interface and pCLAMP 10.0 software (Axon Instruments). Digitalized signals were sampled at 10 kHz and filtered at 2 kHz. Initial fEPSP slopes after tetanic stimulation were normalized to the average baseline fEPSP slope. For statistical analyses, two-way repeated-measures analysis of variance and Sidak’s multiple comparisons test were used on the average of the first 5 minutes and the last 20 minutes of recording after LTP induction.

### Statistical Analysis

Mouse behavioral data are presented as mean ± SEM and as individual data. For behavioral experiments, statistics presented in the figures were based on Student *t* tests. Data were also analyzed using two-way analysis of variance plus Holm-Sidak post hoc analyses with identical results. *p* < .05 was considered significant. *p* > .05 was denoted as nonsignificant. GraphPad Prism 7 software was used to perform statistical analyses and to generate graphical representations of the data.

## Results

### Male but Not Female *Tsc2*^+/−^ Mice Show Deficits in OPR

**Figure 1:**
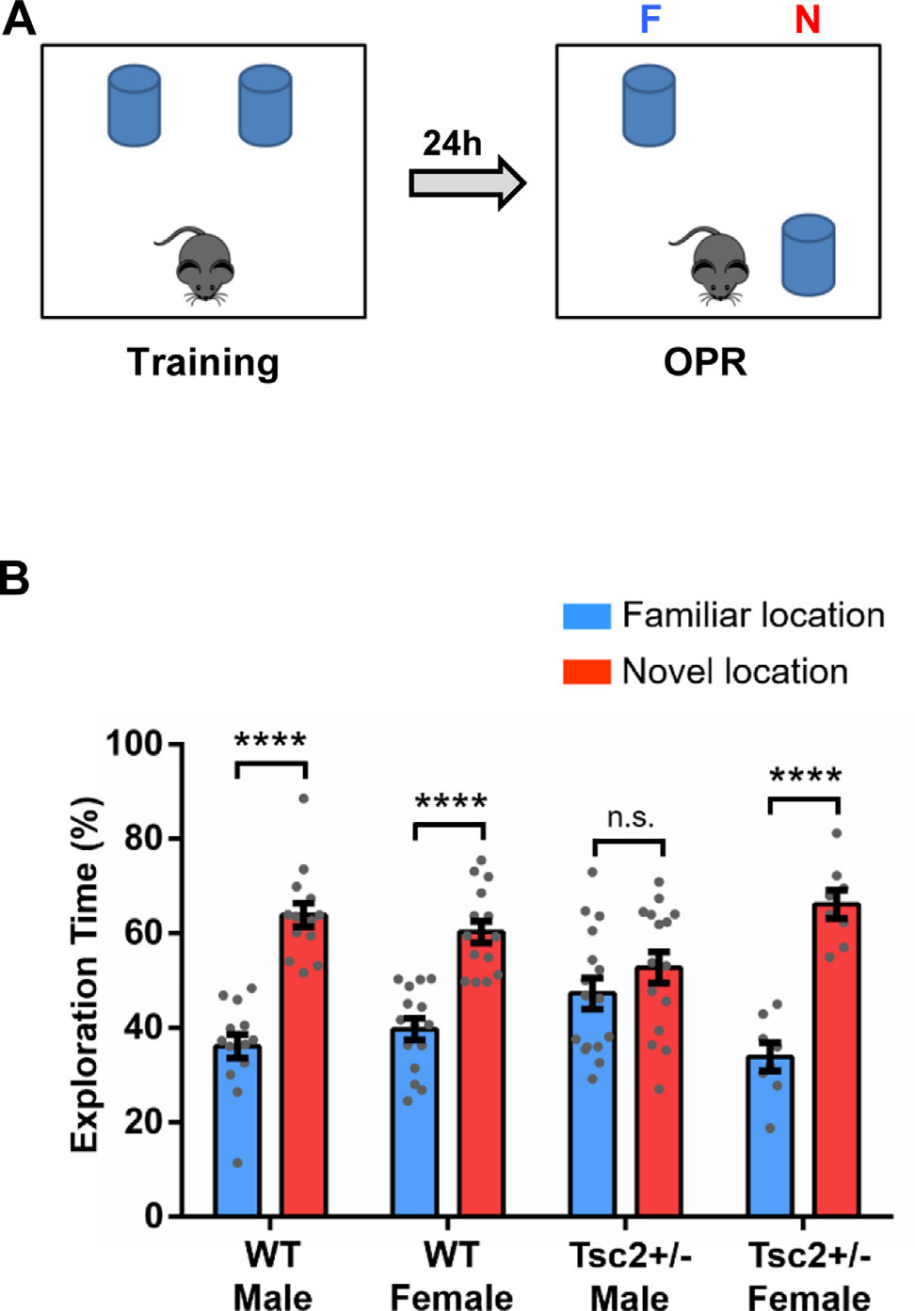
Male but not female *Tsc2*^+/−^ mice show OPR deficits. **(A)** OPR outline. **(B)** Graph shows the percentage of time that mice spent actively exploring the objects in the novel and familiar locations. WT male (*n* = 14; *p* < .0001, *t* = 7.81), WT female (*n* = 15; *p* < .0001, *t* = 6.35), and *Tsc2*^+/−^ female (*n* = 8; *p* < .0001, *t* = 7.59) mice show normal OPR (they explore significantly more the object in the novel location than the object in the familiar location), but *Tsc2*^+/−^ male (*n* = 16; *p* = .25, *t* = 1.16) mice show OPR deficits tested 24 hours after training (show no preference for the object in the novel location). Data represent mean ± SEM as well as individual data. ∗∗∗∗*p* < .0001. F, familiar; OPR, object place recognition; N, novel; n.s., not significant; WT, wild-type.

Male and female *Tsc2*^+/−^ mice and their WT littermates (WT mice) were tested as adults (4–6 months old) in the OPR task (Figure 1A). Male *Tsc2*^+/−^ mice, but not female *Tsc2*^+/−^ or WT mice, showed OPR deficits (no preference for the object in the novel location versus the object in the same location as during training) (Figure 1B). All groups tested (Figure S1A) showed no deficits and equivalent performance in the object recognition memory task (novel object recognition) (24) (Figure S1B), indicating that the OPR deficits of male *Tsc2*^+/−^ mice are not due to deficits in object recognition.

### A Single Adult Treatment With an mTOR Inhibitor (Rapamycin) Can Temporarily Rescue OPR Deficits of Male *Tsc2*^+/−^ Mice

**Figure 2:**
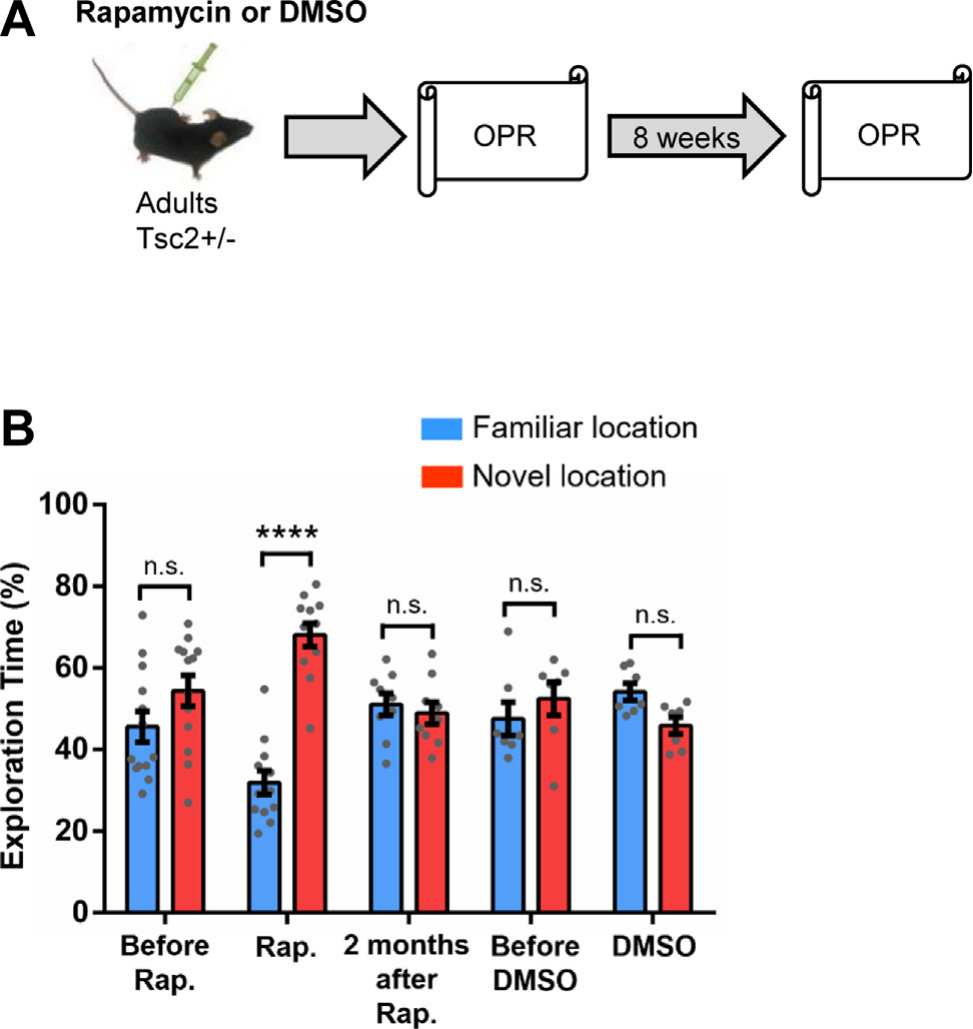
Effects of rapamycin on OPR deficits of male *Tsc2*^+/−^ mice. **(A)** Outline of treatment with rapamycin or DMSO and behavior approach. **(B)***Tsc2*^+/−^ male mice before rapamycin treatment (*n* = 13; *p* = .11, *t* = 1.64) show OPR deficits tested 24 hours after training. *Tsc2*^+/−^ mice during rapamycin treatment (*n* = 12; *p* < .0001, *t* = 8.90) show normal OPR. In contrast, *Tsc2*^+/−^ mice 2 months after rapamycin treatment (*n* = 9; *p* = .58, *t* = 0.55) again show OPR deficits. Data represent mean ± SEM as well as individual data points. ∗∗∗∗*p* < .0001. n.s., not significant; OPR, object place recognition; Rap, rapamycin.

The *Tsc2* gene is known to regulate mTOR (mechanistic target of rapamycin) signaling (27, 28, 29, 30). Accordingly, reductions of TSC2 cause upregulation of mTOR signaling in rodents and humans (23,31). Previously, our laboratory demonstrated that *Tsc2*^+/−^ mice (same genotype/background that we used in this study) have upregulated mTOR signaling (9). The germline mutation that these mice have should affect all cells including the microglia. Previous results showed that an mTOR inhibitor (rapamycin) was capable of reversing multiple phenotypes observed in *Tsc2*^+/−^ mice (9,32,33). Moreover, our laboratory demonstrated that rapamycin treatment effectively reduces hippocampal p-S6 (on Ser235 and Ser236) in both WT and *Tsc2*^+/−^ mice (9). To test whether the inhibition of mTOR signaling in adult male *Tsc2*^+/−^ mice can reverse their OPR deficits, we treated those mice with rapamycin (5 mg/kg) or vehicle (DMSO) as adults (Figure 2A) for 5 days. Before treatment with rapamycin, *Tsc2*^+/−^ mice showed deficits in OPR (Figure 2B), while *Tsc2*^+/−^ mice treated with rapamycin show normal OPR (preference for the object in the novel location) (Figure 2B). However, 2 months after rapamycin treatment, *Tsc2*^+/−^ mice again show OPR deficits (Figure 2B). These results demonstrate the critical role of mTOR signaling in OPR deficits of adult *Tsc2*^+/−^ male mice and show that a 5-day treatment of rapamycin can only temporarily rescue OPR deficits of *Tsc2*^+/−^ male mice.

### Adult Depletion of Microglia Results in the Permanent Rescue of OPR Deficits of Male *Tsc2*^+/−^ Mice

**Figure 3:**
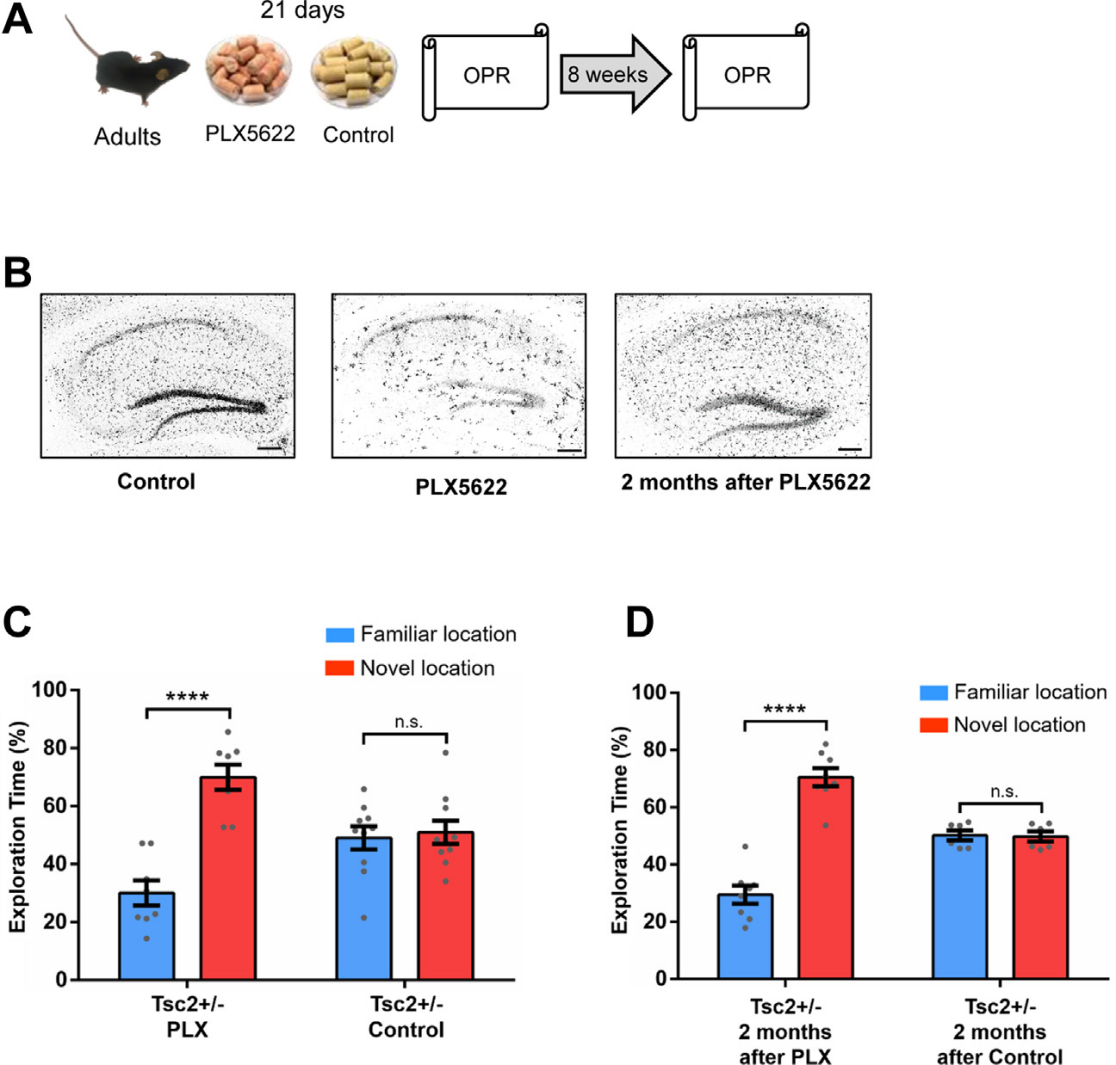
Evidence for a critical role of microglia in OPR memory deficits of male *Tsc2*^+/−^ mice. **(A)** Outline of treatment with PLX, which depletes microglia, or control chow and behavior approach. **(B)** IBA1 immunostaining of *Tsc2*^+/−^ control mice, PLX mice, and mice 2 months after PLX. Treatment with PLX led to elimination of microglia in the whole brain (hippocampus shown as example) compared with the control group (hippocampus shown as example). Two months after PLX, the microglia had repopulated the brain (hippocampus shown as example). **(C)***Tsc2*^+/−^/PLX mice (*n* = 8; *p* < .0001, *t* = 6.50), but not *Tsc2*^+/−^/control mice (*n* = 10; *p* = .73, *t* = 0.33), show normal OPR memory tested 24 hours after training. **(D)***Tsc2*^+/−^ mice 2 months after PLX (*n* = 8; *p* < .0001, *t* = 9.20), but not *Tsc2*^+/−^ mice 2 months after control (*n* = 6; *p* = .86, *t* = 0.17), show normal OPR memory. Data represent mean ± SEM as well as individual data. Scale bar = 200 μm. ∗∗∗∗*p* < .0001. n.s., not significant; OPR, object place recognition; PLX, PLX5622.

Previous results from our laboratory (22) showed that microglia play a critical role in the social memory deficits of *Tsc2*^+/−^ mice given early postnatal immune activation. Therefore, we tested whether depletion of microglia could also reverse OPR deficits of *Tsc2*^+/−^ male mice. Inhibition of CSF1R leads to the depletion of approximately 99% of all microglia brain-wide (34) (Figure 3B). Thus, we treated male *Tsc2*^+/−^ mice with a CSF1R inhibitor (PLX chow) or vehicle control chow (Figure 3A) for 21 days and then tested them for OPR. Treatment with PLX rescued the OPR deficits of male *Tsc2*^+/−^ mice (Figure 3C). Remarkably, we also found that this treatment results in a permanent rescue of OPR in male *Tsc2*^+/−^ mice, because the mice showed normal OPR when they were tested 2 months after microglia depletion (Figure 3D). Histological studies demonstrated that, at this time, microglia had already repopulated the brain of these mice (Figure 3B). These results demonstrate a role for microglia in OPR deficits of male *Tsc2*^+/−^ mice.

As we mentioned above, our laboratory has previously shown that early postnatal immune activation reveals social memory deficits in *Tsc2*^+/−^ mice (*Tsc2*^+/−^ Ep) (22). Thus, we determined whether the depletion of microglia is still capable of rescuing OPR deficits of *Tsc2*^+/−^ Ep adult mice (4–6 months old). Remarkably, treatment with PLX also rescued OPR deficits of male *Tsc2*^+/−^ Ep mice (Figure S2B), and this rescue persisted for at least 2 months (Figure S2C).

### The *Tsc2*^+/−^ Mutation Restricted to Microglia During Development, but Not in Adults, Results in OPR Deficits

**Figure 4:**
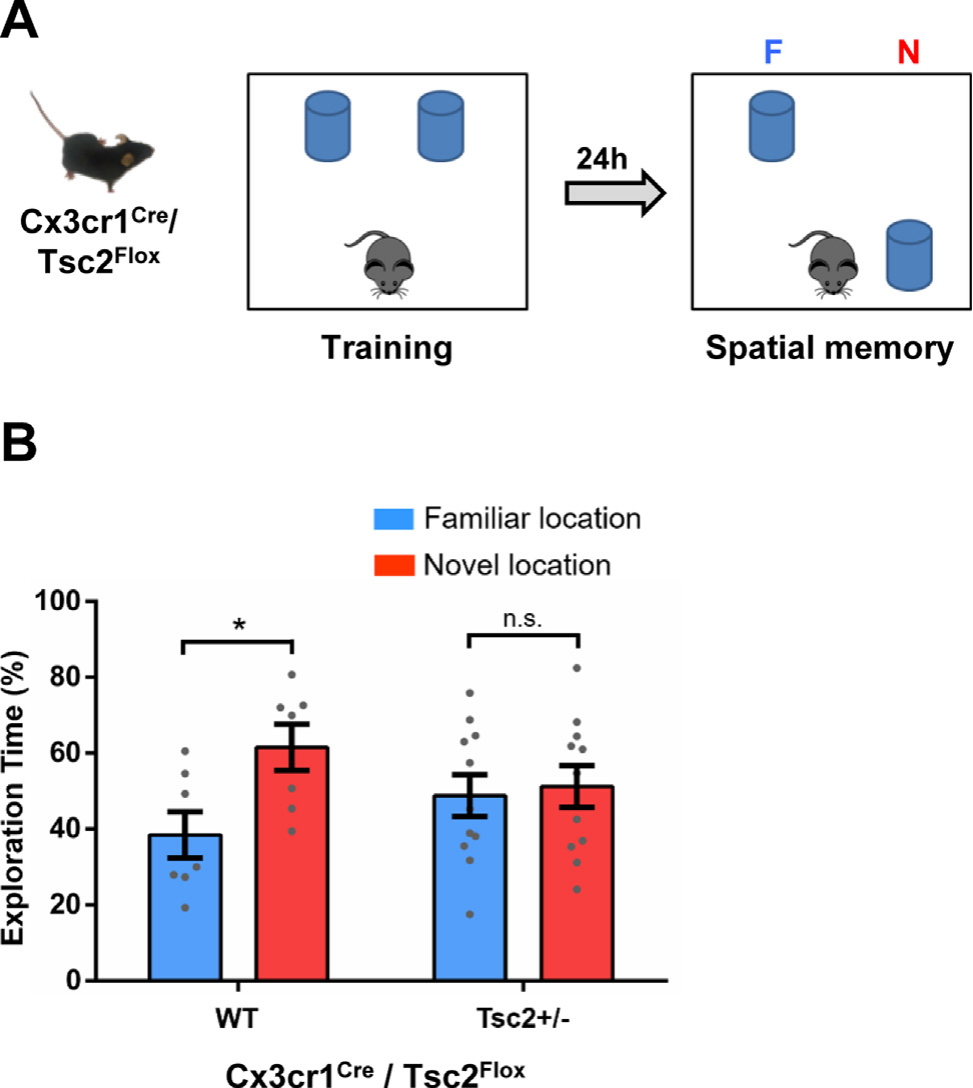
*Tsc2* mutation restricted to microglia during development (but not in adult mice) leads to OPR memory deficits. **(A)** Outline of behavior approach for *Cx3cr1*^Cre^–*Tsc2*^Flox^. **(B)** Male WT (*n* = 7; *p* < .05, *t* = 2.69) mice show normal OPR. In contrast, *Cx3cr1*^Cre^–*Tsc2*^Flox^ (*Tsc2*^+/−^) (*n* = 11; *p* = .76, *t* = 0.3) mice show deficits in OPR tested 24 hours after training. Data represent mean ± SEM as well as values for individual mice. As indicated, in **(B)**, *Tsc2*^+/−^ represents *Cx3cr1*^Cre^–*Tsc2*^Flox^. ∗*p* < .05. F, familiar; N, novel; n.s., not significant; OPR, object place recognition; WT, wild-type.

To test whether the *Tsc2*^+/−^ mutation restricted to the microglia (35) is sufficient to cause OPR deficits, we used a mouse model with a floxed *Tsc2* allele specifically deleted in microglia by a Cre recombinase expressed from a microglia specific promoter (*Cx3cr1*; *Cx3cr1*^Cre^–*Tsc2*^Flox^) (35). We tested those mice as adults (4–6 months old) for OPR (Figure 4A). Similar to germline *Tsc2*^+/−^ male mice, male *Cx3cr1*^Cre^–*Tsc2*^Flox^ mice, but not control mice, showed OPR deficits (Figure 4B).

The results presented above (Figure 3D; Figure S2C), showed that depletion of microglia permanently rescue OPR deficits of male *Tsc2*^+/−^ mice. Therefore, once microglia are depleted in adult mice, the *Tsc2*^+/−^ mutant microglia that repopulate the adult brain of these mice (after PLX treatment in adults) are not able to trigger OPR deficits. This suggests that the *Tsc2* mutation, specifically during development, is critical for OPR deficits of male *Tsc2*^+/−^ mice.

### Microglia Depletion Reverses Abnormal LTP of Male *Tsc2*^+/−^ Mice

**Figure 5:**
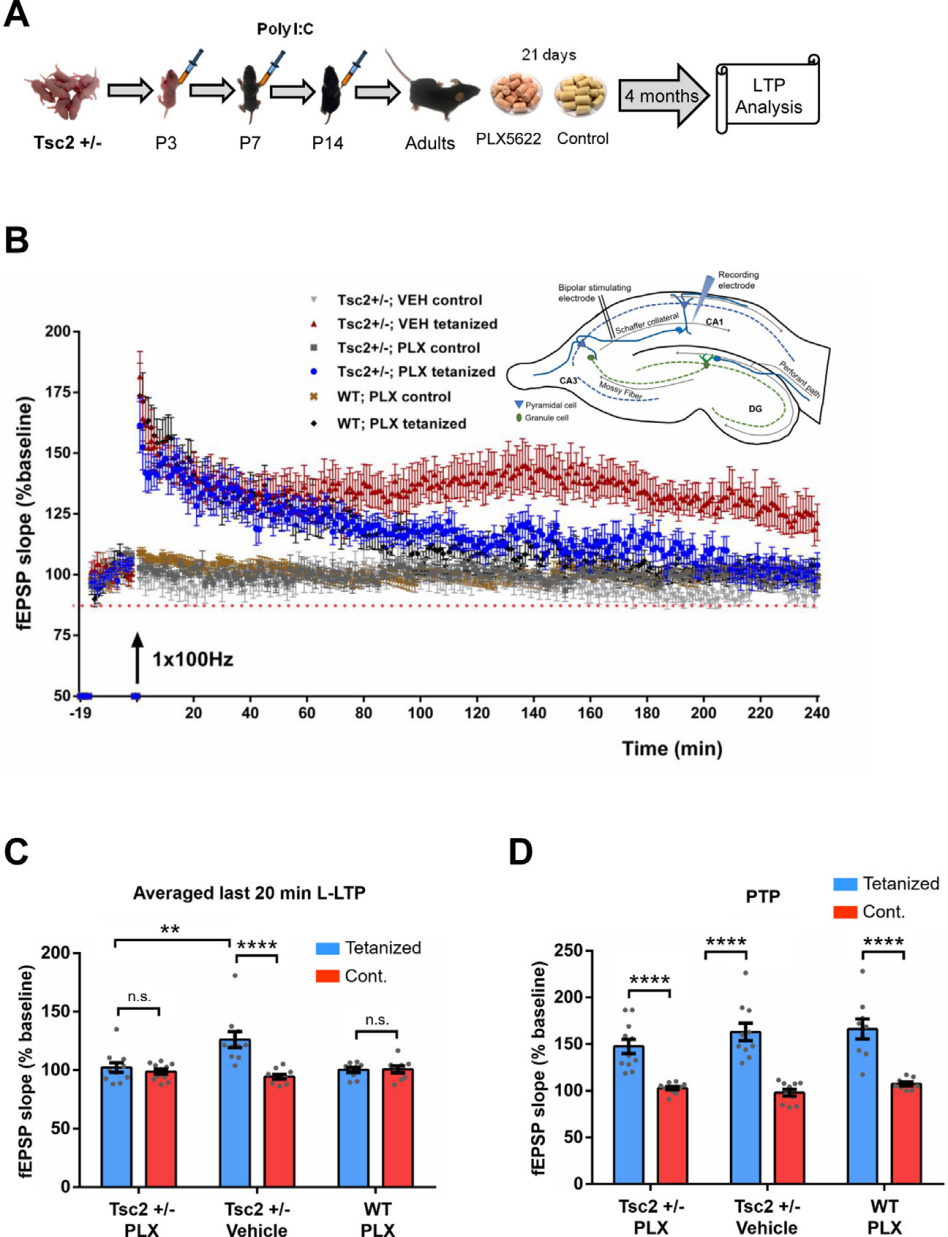
Effects of microglia depletion in the CA1 LTP of male *Tsc2*^+/−^ Ep mice. **(A)** Outline of poly(I:C) injections, treatment with PLX, and analysis approach. **(B)** Initial fEPSP slopes recorded in the CA1 region of hippocampal slices are shown before (baseline, 15 minutes) and following LTP induction (with a 1-second, 100-Hz tetanus delivered at time 0) for the tetanized pathway and for a separate, untetanized pathway (control). Data are plotted in 1-minute blocks and fEPSP slopes are normalized to the average baseline response. Sample traces show responses (5 responses were averaged) during baseline (gray) and during the last 5 minutes of recordings (color). Scale bars = 1 mV and 10 ms. **(C)** Averaged fEPSPs during the last 20 minutes of LTP recording. Regular two-way analysis of variance, pathway × genotype interaction: *F*_2,54_ = 10.51, *p* < .001; Sidak’s multiple comparisons for tetanized vs. control pathway fEPSPs, ∗∗∗∗*p* < .0001 for *Tsc2*^+/−^/VEH group, not significant for other two groups; Tukey’s multiple comparisons for tetanized pathway fEPSPs, ∗∗*p* < .01 for *Tsc2*^+/−^/PLX group vs. *Tsc2*^+/−^/VEH group. **(D)** Averaged PTP fEPSPs (5 minutes) recorded immediately after a 100-Hz tetanus. Regular two-way analysis of variance, pathway factor: *F*_1,54_ = 105.1, *p* < .001; genotype factor: *F*_2,54_ = 1.488, *p* > .05; Sidak’s multiple comparisons for tetanized vs. control pathway, ∗∗∗∗*p* < .0001 for all 3 groups. *Tsc2*^+/−^/VEH slices, *n* = 10 from 6 mice. *Tsc2*^+/−^/PLX slices, *n* = 11 from 7 mice. WT/PLX slices, *n* = 9 slices from 5 mice. All data are shown as mean ± SEM. fEPSP, field excitatory postsynaptic potential; L-LTP, late long-term potentiation; LTP, long-term potentiation; n.s., not significant; P, postnatal day; PLX, PLX5622; Poly I:C, polyinosinic:polycytidylic acid; PTP, post-tetanus potentiation; *Tsc2*^+/−^ Ep, *Tsc2*^+/−^ with early postnatal immune activation; VEH, vehicle; WT, wild-type.

Previous studies suggested that abnormal hippocampal CA1 LTP may underlie the spatial memory deficits of *Tsc2*^+/−^ mice (9,13). The results presented above demonstrated that microglia play a critical role in the OPR deficits of male *Tsc2*^+/−^ mice (Figure 3C, D; Figure S2B, C). We recently demonstrated that poly(I:C) given early postnatally triggers social memory deficits (also hippocampal dependent) in *Tsc2*^+/−^ mice (36). To test if microglia are also responsible for the abnormal LTP of *Tsc2*^+/−^ mice, we treated adult male *Tsc2*^+/−^ Ep mice with PLX or control chow, and 4 months later, we tested their LTP (Figure 5A). We tested *Tsc2*^+/−^ Ep mice, and not *Tsc2*^+/−^ mice, because the hippocampal memory deficits of *Tsc2*^+/−^ Ep mice (which also include social memory deficits) are more severe than those of *Tsc2*^+/−^ mice (36). Remarkably, the results show that depletion of microglia rescued the abnormal LTP of male *Tsc2*^+/−^ Ep mice (Figure 5B–D). These results demonstrate that microglia have a critical role in the abnormal LTP of *Tsc2*^+/−^ mice and suggest that this may contribute to their spatial learning and memory deficits.

### Normal Short-term Memory for OPR in *Tsc2*^+/−^ Male Mice

**Figure 6:**
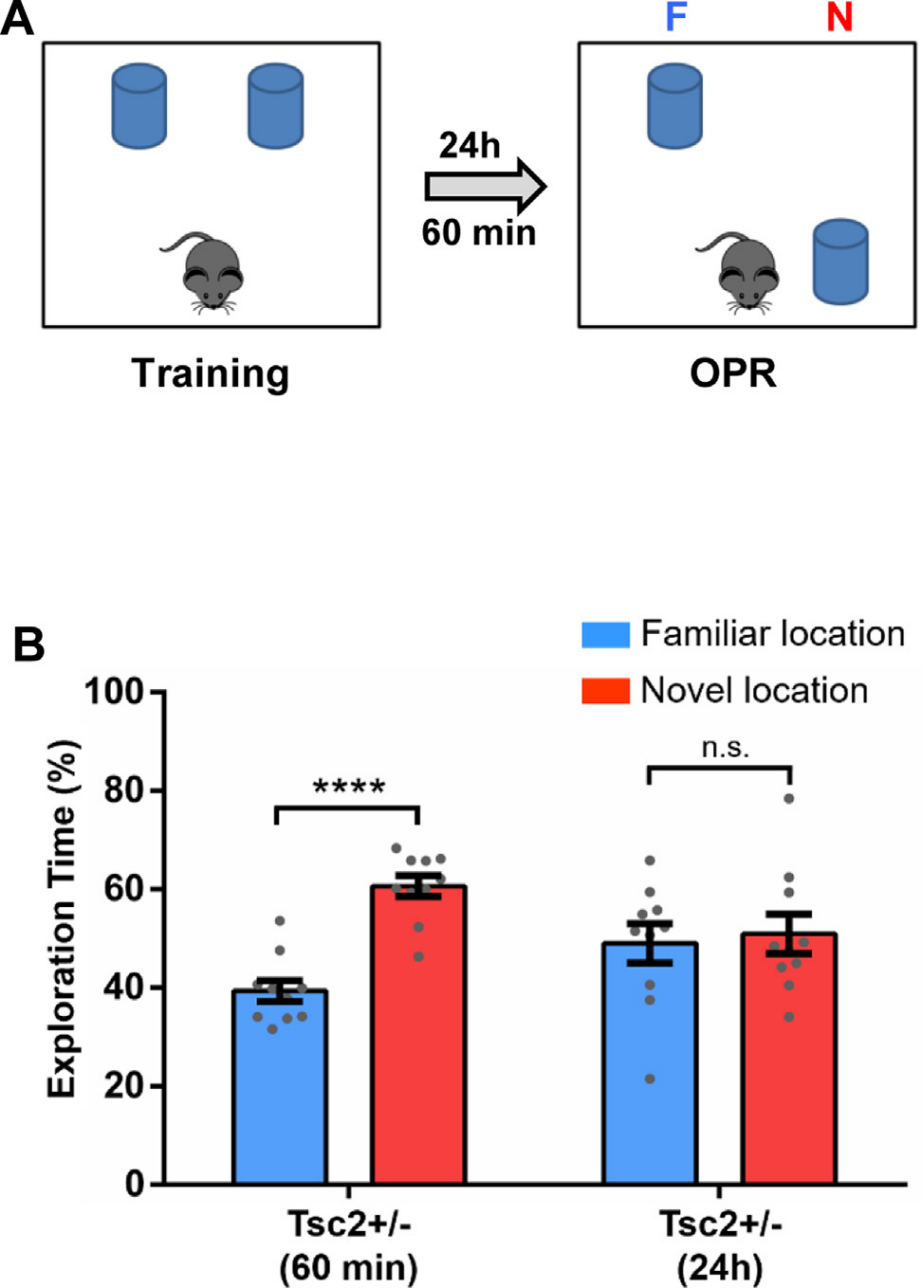
Male *Tsc2*^+/−^ mice show normal OPR memory 60 minutes after training. **(A)** Outline of behavior approach. **(B)** Graph shows the percentage of time the mice spent actively exploring the objects in the novel and familiar locations. *Tsc2*^+/−^ mice tested 60 minutes after training (*Tsc2*^+/−^/60 min; *n* = 10; *p* < .0001, *t* = 6.97) show normal OPR (they explore the object in the novel location significantly more than the object in the familiar location), but *Tsc2*^+/−^ mice tested 24 hours after training (*Tsc2*^+/−^/24h; *n* = 10; *p* = .73, *t* = 0.33) show OPR deficits (show no preference for the object in the novel location). Data represent mean ± SEM as well as individual data. ∗∗∗∗*p* < .0001. Cont. control; F, familiar; N, novel; n.s., not significant; OPR, object place recognition.

Prior results (9), as well as the LTP experiments described above (Figure 5B–D), revealed that *Tsc2*^+/−^ mice (with or without early postnatal immune activation) have normal LTP tested 60 minutes after induction, whereas LTP tested 3 hours after induction was abnormal. In the experiments described above (Figure 1), OPR memory was tested 24 hours after training. In addition, TSC2 affects mTOR signaling (23,31), and this signaling pathway has been implicated in memory consolidation (37). Thus, we next tested whether adult *Tsc2*^+/−^ male mice have deficits in OPR at a time both before memory consolidation (60 minutes after OPR training) and when their LTP is normal (Figure 5B–D). Consistent with the idea that abnormal LTP may contribute to OPR memory deficits of *Tsc2*^+/−^ male mice, we found that these mutant mice show normal OPR when tested 60 minutes after training (Figure 6B). When tested 24 hours after training, *Tsc2*^+/−^ male mice once again revealed OPR deficits (Figure 6B). These results support the hypothesis that the abnormal LTP of *Tsc2*^+/−^ mice contributes to their OPR memory deficits.

### *Ifnar1* Knockout (*Ifnar1*^−/−^) Prevents OPR Deficits in Male *Tsc2*^+/−^ Mice

**Figure 7:**
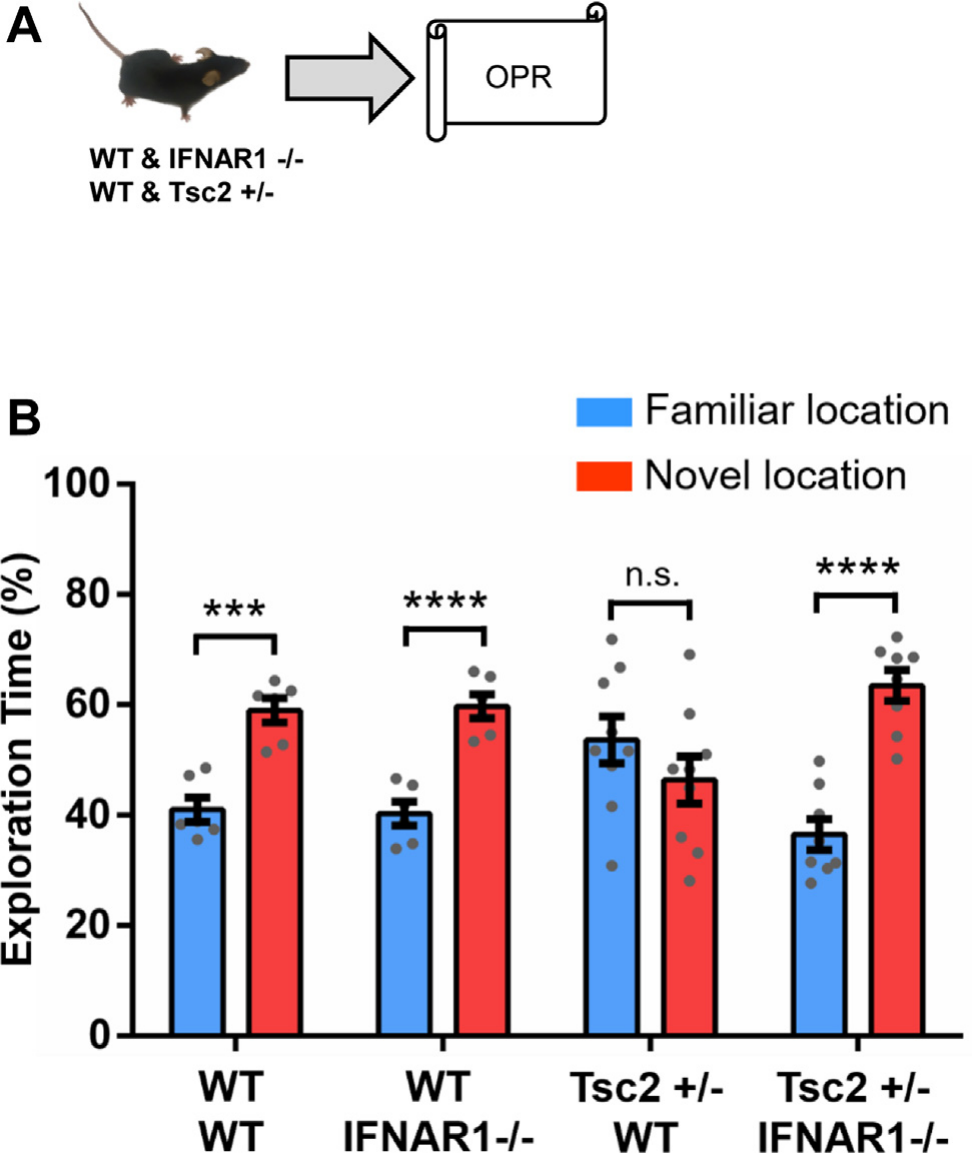
Effects of the null homozygous mutation of type I IFN receptor (*Ifnar1*^−/−^) in *Tsc2*^+/−^ OPR deficits. **(A)** Behavior approach. **(B)***Tsc2*^+/−^ mice (*n* = 9; *p* = .24, *t* = 1.19) show OPR deficits tested 24 hours after training. WT (*n* = 6; *p* < .001, *t* = 5.71), *Ifnar1*^−/−^ (*n* = 6; *p* < .0001, *t* = 6.34), and *Tsc2*^+/−^/*Ifnar1*^−/−^ (*n* = 8; *p* < .0001, *t* = 6.78) mice show normal OPR. Data represent mean ± SEM as well as values for individual mice. ∗∗∗*p* < .001, ∗∗∗∗*p* < .0001. IFN, interferon; n.s., not significant; OPR, object place recognition; WT, wild-type.

In our previous work, we determined that type I IFN plays a critical role in the social memory deficits of male *Tsc2*^+/−^ mice (22). Here, we determined whether type I IFN signaling also has a role in OPR deficits of male *Tsc2*^+/−^ mice (Figure 7A). Remarkably, although male *Tsc2*^+/−^ mice showed robust OPR deficits (Figure 7B; as we observed in Figure 1B), male *Tsc2*^+/−^ mice with a null mutation for the *Ifnar1* gene (*Tsc2*^+/−^/*Ifnar1*^−/−^) showed normal OPR (Figure 7B). These results demonstrate the role of type I IFN signaling in the OPR deficits of male *Tsc2*^+/−^ mice.

## Discussion

Although previous studies focused on the role of neuronal deficits in the memory phenotypes of rodent models of TSC (14,15), the results presented here demonstrate a role for microglia in these memory deficits. In addition to rescuing their OPR deficits, we show that depletion of microglia also reversed the abnormal LTP of *Tsc2*^+/−^ mice.

TSC-associated neuropsychiatric disorders include autism, intellectual disability, and a range of other psychiatric and behavioral symptoms (3, 4, 5, 6, 7, 8, 9,38). In addition, epilepsy affects up to 80% of patients with TSC, with severe seizures that can be treatment resistant and that could contribute to their behavioral abnormalities (39). While neuronal mechanisms have played a key role in explanations of autism and intellectual disability phenotypes of patients with TSC (14,15), glia have been increasingly recognized to play important roles in TSC (40). For example, there is growing evidence for the role of astrocytes, microglia, and oligodendrocytes in the pathophysiology of epilepsy in TSC (40). Our previous results (22), as well as those presented here, also suggest that glial cells, particularly microglia, have a role in the cognitive phenotypes of TSC and that targeting glial mechanisms could be a viable therapeutic approach for addressing their cognitive deficits.

Pathology studies of tubers dissected from epileptic patients with TSC revealed evidence of microglial activation (41,42). However, seizures are common in patients with TSC and may cause microglia activation themselves (43), thus making it difficult to determine whether microglia activation is a consequence of other pathological causes in TSC or a contributing cause of TSC phenotypes. For example, previous studies showed that mice with an astrocyte-specific *Tsc1* mutation showed evidence of microglia activation, as well as increased microglia size and number (44). The results presented here make a strong case that abnormalities in microglia may not simply be a consequence of changes in other cell types involved in the pathophysiology of TSC, such as astrocytes and neurons, because we showed that the *Tsc2*^+/−^ mutation restricted to microglia recreated the hippocampal memory deficits associated with animal models of TSC (7,9,36,45,46). In addition, we also showed that the hippocampal CA1 LTP abnormalities and OPR deficits of *Tsc2*^+/−^ mice could be reversed by a manipulation that depleted most microglia from the brain of these mice. We demonstrated that a null mutation (*Ifnar1*^−/−^) of a key signaling pathway in microglia (47) also prevented the hippocampal-dependent memory deficits of these mice. Previous RNA sequencing studies in patients with autism spectrum disorder (48) pointed to a lasting upregulation of microglia and IFN response pathways, suggesting that our findings with an animal model of TSC may be more generally significant.

We previously proposed (22) that *Tsc2* haploinsufficiency (*Tsc2*^+/−^) leads to abnormally elevated mTOR signaling (23,31) and, consequently, to increased production of type I IFN. Elevated IFN levels result in overactivation of IFNAR1, which in turn further activates mTOR signaling and triggers even more IFN production. We propose that this cycle is perpetuated in microglia, and it is responsible for the OPR and LTP deficits of male *Tsc2*^+/−^ mice.

Our results suggest that the *Tsc2*^+/−^ mutation during development is critical for OPR deficits: first, we showed that rapamycin treatment during early postnatal development (at P3, P7, and P14) can prevent OPR deficits of *Tsc2*^+/−^ mice (tested ∼4 months after this treatment) (Figure S3), which suggests that this developmental period is critical for OPR deficits of male *Tsc2*^+/−^ mice. Second, rapamycin treatment specifically in adult *Tsc2*^+/−^ mice rescues their OPR deficits (Figure 2B). Resident microglia are long lived, with a median lifetime of well over 15 months (49). Thus, approximately half of these cells survive the entire mouse life span. Therefore, changes in these cells during development could contribute to OPR deficits of *Tsc2*^+/−^ mice. Finally, depletion of microglia in adult *Tsc2*^+/−^ mice rescues OPR deficits (Figure 3C), and the rescue persists after new microglia repopulate the brain in adults (Figure 3D), which indicates that new mutant microglia do not trigger OPR deficits in *Tsc2*^+/−^ mice, suggesting that changes in development are critical.

Altogether, the results presented here demonstrate that microglia and type I IFN signaling have a key role in the electrophysiological and memory phenotypes of an animal model of TSC, a result consistent with the hypothesis that abnormal type I IFN signaling in microglia is responsible for abnormal LTP and for hippocampal memory deficits in *Tsc2*^+/−^ mice. These phenotypes can be reversed by depletion of microglia, a result that suggests that therapeutic strategies targeting this cell type may be a viable strategy to address cognitive impairments in TSC.

## Supplementary Material

Supplementary Material

Key Resources Table
