# Supplementary material for: Role of Type I Interferon Signaling and Microglia in the Abnormal Long-term Potentiation and Object Place Recognition Deficits of Male Mice With a Mutation of the Tuberous Sclerosis 2 Gene
Source: Biol Psychiatry Glob Open Sci. 2022 Apr 14;3(3):451–9. doi: 10.1016/j.bpsgos.2022.03.015 (PMC10382699; doi:10.1016/j.bpsgos.2022.03.015)
Supplement: Supplementary Material [file mmc1.pdf]

Role of Type I Interferon Signaling and Microglia in the Abnormal Long-Term Potentiation and Object Place Recognition Deficits of Male Mice With a Mutation of the Tuberous Sclerosis 2 Gene

Supplementary Information

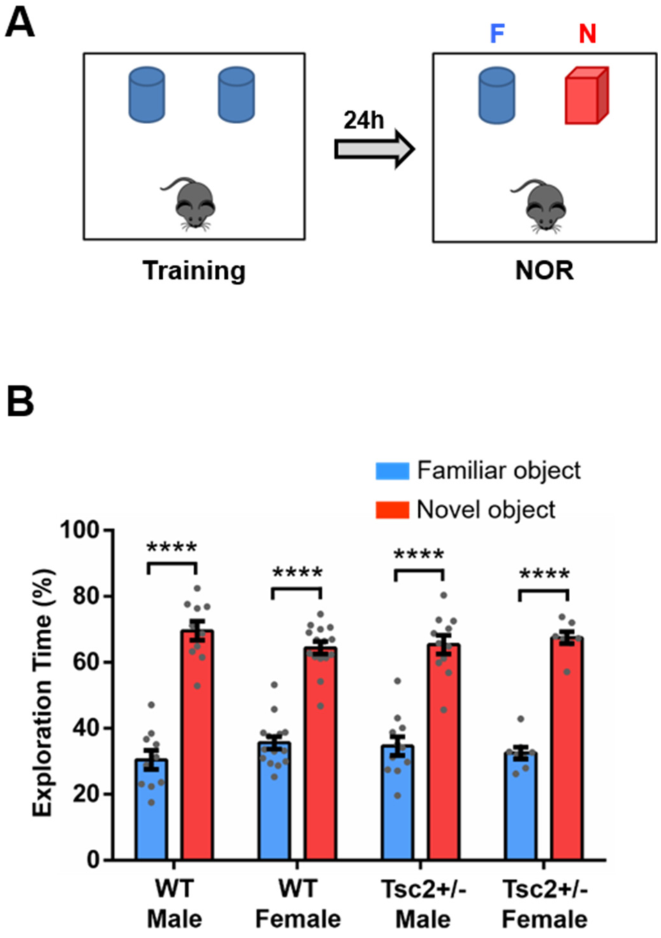

Figure S1. Tsc2$^{+/-}$ male mice show normal object memory.

(**A**) Outline of behavior approach. (**B**) All groups, WT/male (n=10; P<0.0001, t=9.64), WT/female (n=15; P<0.0001, t=11.09), Tsc2$^{+/-}$/male (n=11; P<0.0001, t=7.68) and Tsc2$^{+/-}$/female (n=8; P<0.0001, t=14.07) mice show normal object memory (they spent significantly more time exploring the novel object than the familiar object). Data represent means ± SEM as well as individual data.

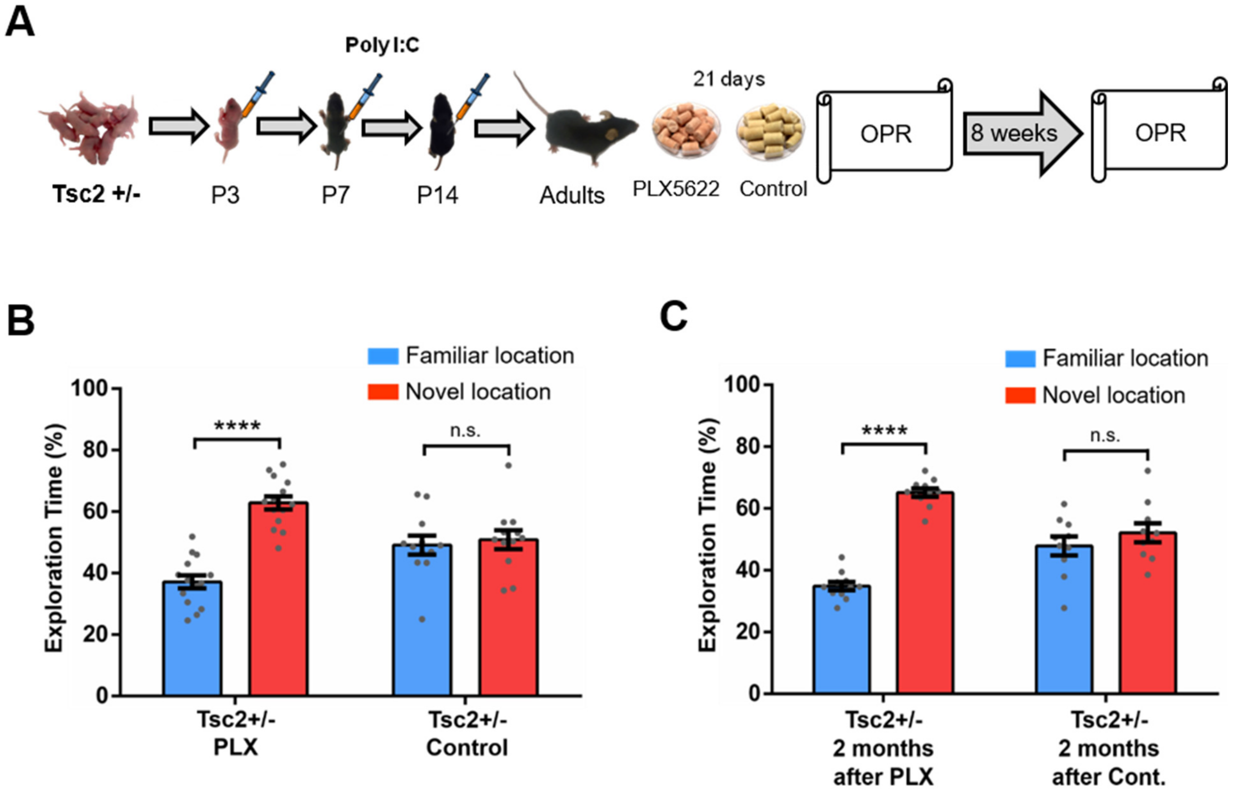

Figure S2. Depletion of microglia rescues the OPR deficits of male Tsc2$^{+/-}$ Ep mice.

(**A**) Outline of treatment with PLX5622 (PLX; depletes microglia) or Control chow and behavior approach. (**B**) Tsc2$^{+/-}$/PLX mice (n=14; P<0.0001, t=8.44) but not Tsc2$^{+/-}$/Control (n=12; P=0.68, t=0.40) mice, show normal OPR. (**C**) Tsc2$^{+/-}$ mice two months after PLX (n=11; P<0.0001, t=16.2), but not Tsc2$^{+/-}$ mice 2 months after Control (n=10; P=0.34, t=0.97), show normal OPR memory. Data represent mean ± SEM as well as individual data.

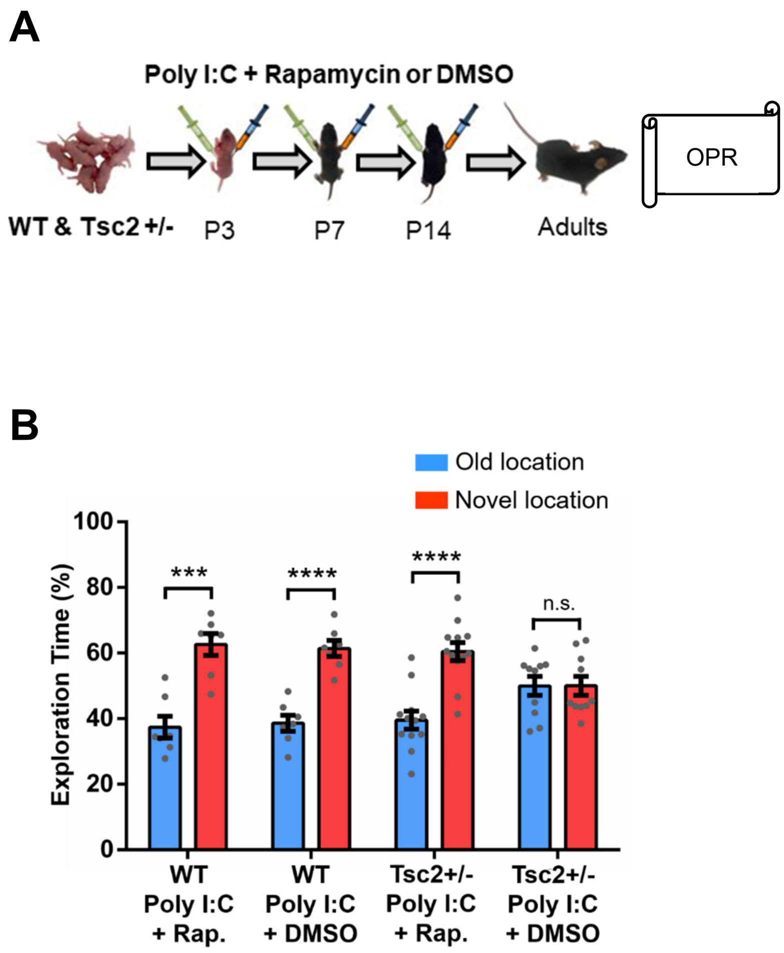

Figure S3. Effects of rapamycin early post-natally in the OPR deficits of male $Tsc2^{+/-}$ Ep mice.

(**A**) Outline of the timeline of injections of Poly I:C and treatment with rapamycin (Rap) or DMSO (control). (**G**) WT/Poly I:C+Rap (n=7; P<0.001, t=5.32), WT/Poly I:C+DMSO (n=7; P<0.0001, t=6.56) and $Tsc2^{+/-}$/Poly I:C+Rap (n=12; P<0.0001, t=5.41) mice show normal OPR. $Tsc2^{+/-}$/Poly I:C+DMSO (n=10; P=0.99, t=0.001) mice show an OPR deficit. Rap: rapamycin. Data represent means ± SEM as well as individual data points.
